# A Fly on the Wall: How Stress Response Systems Can Sense and Respond to Damage to Peptidoglycan

**DOI:** 10.3389/fcimb.2019.00380

**Published:** 2019-11-13

**Authors:** Antoine Delhaye, Jean-François Collet, Géraldine Laloux

**Affiliations:** ^1^de Duve Institute, UCLouvain, Brussels, Belgium; ^2^Walloon Excellence in Life Sciences and Biotechnology (WELBIO), Brussels, Belgium

**Keywords:** stress response, ESRS, cell wall, Cpx, RCS, Psp, sigmaE, BAE

## Abstract

The envelope of Gram-negative bacteria is critical for survival across a wide range of environmental conditions. The inner membrane, the periplasm and the outer membrane form a complex compartment, home to many essential processes. Hence, constant monitoring by envelope stress response systems ensure correct biogenesis of the envelope and maintain its homeostasis. Inside the periplasm, the cell wall, made of peptidoglycan, has been under the spotlight for its critical role in bacterial growth as well as being the target of many antibiotics. While much research is centered around understanding the role of the many enzymes involved in synthesizing the cell wall, much less is known about how the cell can detect perturbations of this assembly process, and how it is regulated during stress. In this review, we explore the current knowledge of cell wall defects sensing by stress response systems, mainly in the model bacterium *Escherichia coli*. We also discuss how these systems can respond to cell wall perturbations to increase fitness, and what implications this has on cell wall regulation.

## The Cell Wall of Gram-Negative Bacteria

**Figure 1:**
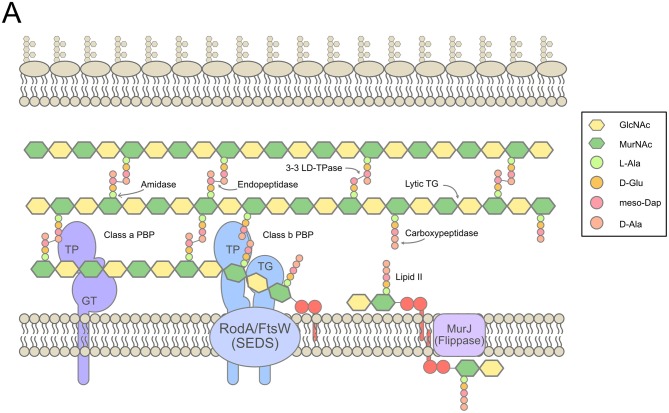
Overview of cell wall synthesis in *E. coli*. **(A)**. The synthesis of PG in the periplasm from lipid II precursors in the cytoplasm, with indications of the main synthetic, and lytic activities involved. Figure inspired by Typas et al. (2012), Cho S.H. et al. (2014), and Zhao et al. (2017).

In the environment, bacteria face an ever-changing range of conditions to which they have to adapt in order to survive and thrive. To overcome the many challenges that they face, Gram-negative bacteria have evolved a complex envelope made out of two membranes, the inner membrane (IM) and the outer membrane (OM) surrounding a soluble chamber, the periplasm. The OM is asymmetric, composed of phospholipids in the inner leaflet and lipopolysaccharides (LPS) in the outer leaflet (Silhavy et al., 2010). In the periplasm lies the cell wall, the determinant of cell shape, and essential for resistance to osmotic stress (Höltje, 1998; Vollmer et al., 2008). The cell wall is composed of a single-layered biopolymer, the peptidoglycan (PG), composed of repeating units of a disaccharide (N-acetylglucosamine-N-acetylmuramic acid, or GlcNac-MurNac) crosslinked with short peptides, forming a mesh-like structure (the main architecture of the PG and its assembly are summarized in Figure 1). The synthesis of PG proceeds in 3 major steps, all of which can be inhibited by antibiotics (Zhao et al., 2017): (1) the generation of the key intermediate lipid II, the lipid-linked disaccharide-pentapeptide precursor, in the cytoplasm; (2) the translocation of lipid II across the cytoplasmic membrane; and (3) the assembly of the cell wall in the periplasm (Typas et al., 2012; Ruiz, 2015). During growth, elongation is the process by which cells increase their size, and division is the process by which one bacterial cell separates into two daughter cells. In *E. coli* and other Gram-negative bacteria, both these processes rely on complex PG remodeling involving both PG synthesis and PG degradation (van Teeffelen and Renner, 2018). PG synthesis requires the polymerization of new glycan strands by transglycosylases and the crosslinking of their peptide side-chains by transpeptidases. To this end, multiple PG synthases are required. Monofunctional glycosyltransferases of the shape, elongation, division and sporulation (SEDS) family polymerize GlcNac-MurNac disaccharides from lipid II subunits into long glycan strands. These strands are crosslinked together mostly between the fourth (D-ala) and the third (diaminopimelic acid, DAP) amino acid of their peptide side chains, resulting in 4–3 D,D crosslinks whose formation is catalyzed by Penicillin-Binding Proteins (PBPs). The broad family of PBPs consists of two different classes: the class B PBPs are monofunctional and can only carry out the transpeptidase reaction, while the bifunctional class A PBPs also exhibit a transglycosylase activity. The main class A PBPs in *E. coli* are PBP1a and PBP1b. Neither is essential in normal conditions, but a double mutant lacking both is nonviable (Sauvage et al., 2008). In addition to the 4–3 D,D crosslinks, non-canonical 3–3 L,D crosslinks between two DAP residues of peptide side chains are synthesized by L,D-transpeptidases that are mostly active during stationary phase (Pisabarro et al., 1985; Magnet et al., 2007, 2008). These 3–3 crosslinks are also required when defects in the LPS transport pathway occur, to strengthen the PG and avoid lysis (Morè et al., 2019). Additionally, cell wall homeostasis during both elongation and division requires enzymes that digest the PG to allow the insertion of newly synthesized material (Uehara and Bernhardt, 2011). PG fragments (muropeptides) are thus continually extracted from the PG mesh by the action of lytic transglycosylases and endopeptidases, transported back to the cytoplasm through a permease, and recycled predominantly as precursors (although they can be used as an energy source as well) (Park and Uehara, 2008), making the cell wall a highly adaptable entity. In fact, there is mounting evidence that cell wall synthesis is adapted depending on the extracellular environment. Indeed, the activity of cell wall modifying enzymes (such as *E. coli* PBP6b, MltA or *Salmonella* Typhimurium PBP3) changes depending on the chemical properties (pH) of the environment (van Straaten et al., 2005; Peters et al., 2016; Castanheira et al., 2017). A striking example is the requirement of PBP1a for maximal fitness in alkaline conditions and of PBP1b under acidic conditions (Mueller et al., 2019), consistent with the idea that PG synthesis machinery and the general structure of the PG itself change with the environmental conditions to optimize fitness (Pazos et al., 2017).

**Figure 2:**
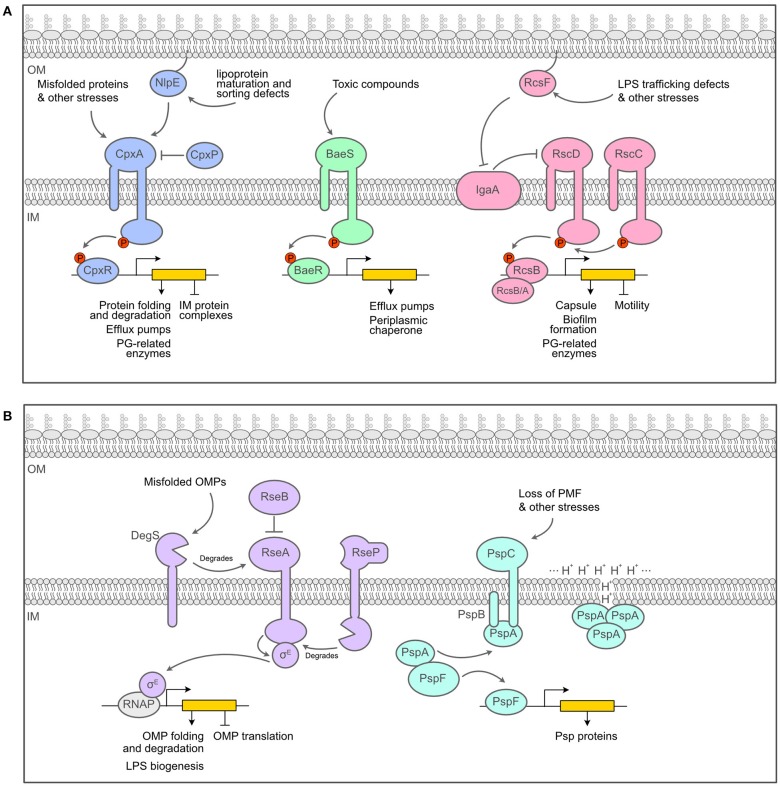
Overview of the major envelope stress response systems of *E. coli*. **(A)**. Schematics of the two-component systems of *E. coli* that act as ESRS, the Cpx, Bae and Rcs systems. **(B)** Schematics of alternative response systems that act as ESRS, the σ^E^ and the Psp responses. Figure inspired by Guest et al. (2017) and Mitchell and Silhavy (2019).

Furthermore, the cell wall is not an isolated entity: it must be constructed and remodeled within the envelope, a compartment home to many delicate and essential processes. The biogenesis of the envelope is a never-ending ballet in which membrane-anchored lipoproteins, integral membrane proteins, β-barrels (outer membrane proteins, OMPs), phospholipids and LPS have to be correctly sorted, transported and inserted in the right membrane (Silhavy et al., 2010; Rollauer et al., 2015; Okuda et al., 2016; Szewczyk and Collet, 2016). Elongation and division of the cell wall must happen without any loss of integrity and in exquisite coordination with both membranes (Gray et al., 2015). It is thus very important for the cell to monitor the state of the envelope to avoid lethal prejudice following changes in the environment, such as variations in pH and osmolarity or the use of antibiotics. Constant surveillance by envelope stress response systems (ESRS) is necessary: these systems transduce distress signals from the envelope, across the IM, into the cytoplasm in order to elicit a reaction to damages in the envelope by modifying gene expression. One of the objectives of this review is to gather evidence that ESRS can monitor cell wall related processes and react to potential problems. Therefore, in the next section, we briefly detail the major ESRS of *E. coli*, schematized in Figure 2.

## Signal Transduction Systems

Two-component systems (TCS) are a universal solution employed to transduce signals across membranes. An archetypal envelope-associated TCS relies on a membrane-embedded sensor histidine kinase and a cytoplasmic response regulator. Upon activation by a specific signal, the histidine kinase autophosphorylates, then transfers the phosphoryl group to the response regulator, which becomes active and proficient for DNA binding to regulate the expression of a particular set of genes (Zschiedrich et al., 2016). We will now briefly introduce the main TCS that are involved in sensing and responding to envelope defects in *E. coli*, i.e., the Cpx, Bae and Rcs responses (Figure 2A).

The Cpx pathway is a classical TCS, consisting of the histidine kinase CpxA and the response regulator CpxR, with two accessory proteins: CpxP, which is a negative regulator of the response, and NlpE, an OM lipoprotein involved in the sensing of lipoprotein maturation and sorting defects (Delhaye et al., 2019; May et al., 2019). Cpx is usually seen as an envelope quality control system detecting the presence of misfolded proteins in the periplasm (Raivio and Silhavy, 1997; Hunke et al., 2011) and activating the expression of folding and degradation factors in response (chaperones and proteases) (Pogliano et al., 1997; Raivio et al., 2013; Surmann et al., 2016). Because of its broad role in protein maintenance and repair, Cpx is considered to be one of the main ESRS. Another classical TCS that has been pegged as an ESRS is the Bae system, composed of the histidine kinase BaeS and the response regulator BaeR (Raffa and Raivio, 2002). Bae mainly regulates the expression of multidrug efflux pumps as well as the expression of *spy*, encoding a periplasmic chaperone (Leblanc et al., 2011; Quan et al., 2011). In contrast to the Cpx and Bae pathways, the Rcs phosphorelay exhibits more complexity (Wall et al., 2018). Instead of the signal being transferred directly from the IM sensor histidine kinase RcsC to the response regulator RcsB, it must first transit through another transmembrane protein at the IM, RcsD (Takeda et al., 2001). RcsD and RcsC are maintained in an inactive state by IgaA, an IM inhibitor of the phosphorelay (Cho S.H. et al., 2014; Hussein et al., 2018). When active, the response regulator RcsB can bind either to itself or to other regulators, such as RcsA to control the expression of distinct sets of genes (Wall et al., 2018). Most Rcs-inducing cues require the presence of an OM lipoprotein, RcsF, for transducing the signal across the periplasm (reviewed in Laloux and Collet, 2017). In inducing conditions, RcsF interacts with IgaA, which relieves its inhibiting effect on Rcs and turns the system on Cho S.H. et al. (2014) and Hussein et al. (2018). The targets of the Rcs phosphorelay include genes involved in important cell surface structures such as flagella, LPS and fimbriae as well as acid resistance and virulence (Bury-Moné et al., 2009; Clarke, 2010). Of note, the expression of the colanic acid capsular polysaccharide genes, i.e., the capsule or *cps* genes which will be mentioned further in this review, is dependent on Rcs and the RcsA/RcsB heterodimer.

There are at least two other systems that monitor the state of the envelope of *E. coli* and that do not rely on a TCS machinery. We introduce these systems here, namely the σ^E^-dependent signaling cascade and the phage shock response (Psp) (Figure 2B). σ^E^ is an alternative sigma factor that is normally sequestered on the cytoplasmic side of the IM by the membrane-bound anti-sigma factor RseA. Under inducing conditions, a cascade of proteolytic reaction degrades RseA and releases σ^E^ in the cytoplasm (Ades et al., 1999). σ^E^-bound RNA polymerase then promotes transcription of genes encoding periplasmic chaperones involved in the transport of unfolded β-barrels across the periplasm, components of the β-barrel assembly machinery (BAM) required for β-barrel insertion in the OM, and proteins involved in LPS assembly (Rhodius et al., 2005; Grabowicz and Silhavy, 2016). On the other hand, the Psp response is induced by many signals, all having in common the fact that they result in severe IM perturbation and disrupt the proton motive force (PMF) (Brissette et al., 1990). During non-inducing conditions, PspF, the transcriptional regulator of the Psp response, is bound to PspA in the cytoplasm, which prevents it from regulating transcription. When induced, the IM proteins PspB and PspC, which are thought to be the sensors of the system, bind PspA, thus freeing PspF to activate the transcription of the *psp*A operon (Darwin, 2005). PspA is also able to bind membrane phospholipids and repair proton leakage of the damaged membranes that set off the response in the first place (Kleerebezem et al., 1996; Kobayashi et al., 2007). Overall, the Psp response seems to help maintaining PMF and thus the energy state of the cell during various envelope stresses such as growth in alkaline pH or bile salts while in the stationary phase (Joly et al., 2010).

Together the Cpx, Bae, Rcs, σ^E^, and Psp systems are usually considered to be the main ESRS of *E. coli*, the “watchdogs of the envelope” (Ruiz and Silhavy, 2005). Important envelope biogenesis processes have been described to be monitored by these systems. For instance, the σ^E^ response directly reacts to problems in the assembly of β-barrels: unfolded OMPs are sensed by the essential IM serine protease DegS, which recognizes and binds a motif in their C-terminal sequence. Binding activates DegS (Walsh et al., 2003), which is then able to degrade RseA and start the proteolytic cascade activating the σ^E^ response, relieving the initial stress. Such an elegant mechanism, in which failures in a process are sensed directly and subsequently activate a response that deals with the damage, has yet to be described for the synthesis and maintenance of the cell wall of *E. coli*. Indeed, while cell wall synthesis and its inhibition by antibiotics have been the subject of a vast amount of research, very little is known about how the cell senses and responds to damage to the PG, or even alter PG composition as a means of adaptation to a dynamic environment. In this review, we will cover the sensing of PG defects by the main ESRS presented above, focusing on *E. coli* but also pointing to some insights obtained with other Gram-negative bacterial species. We will also assess how these systems can help the cell to survive attacks to their cell wall and how other signal transduction pathways, which were not previously thought to be specifically related to envelope stress sensing, can also detect and react to the loss of PG integrity. Table 1 summarizes the different types of PG stress and responses that we are reviewing here.

**Table 1:** List of PG stresses, their effect on stress responses, and the benefits of stress responses on the overall fitness of the cells.

**Source of PG stress**	**Target**	**Stress response activated**	**Evidence**	**Benefits for the cell**
**BETA-LACTAMS**				
A22	MreB	Rcs	P*rprA-lacZ* induction (Cho H. et al., 2014), P*cpxP-lacZ* induction (Delhaye et al., 2016)	
Cephaloridine	PBPB1a	Rcs	*cps* induction (Sailer et al., 2003)	/
Mecillinam	PBP2	Rcs, Cpx, SigmaE	Rcs, Cpx, and sigmaE regulons (microarray), P*rprA-lacZ* induction (Laubacher and Ades, 2008), P*cpxP-lacZ* induction (Delhaye et al., 2016)	Δ*rcsF* and Δ*rcsB* more sensitive than WT, constitutive activation of Rcs leads to enhanced fitness (Laubacher and Ades, 2008), Δ*cpxR* more sensitive than WT, constitutive activation of Cpx leads to enhanced fitness (Delhaye et al., 2016)
Aztreonam	PBP3	Rcs	*wcaE* (Rcs regulon) induction (microarray) (Arends and Weiss, 2004)	/
Cephalexin	PBP3	Cpx, Dpi	P*cpxP-lacZ* induction (Delhaye et al., 2016), *dpiBA* operon induction (Miller et al., 2004)	Δ*cpxR* is slightly more sensitive than WT (Delhaye et al., 2016)
Pipericillin	PBP3	Dpi	*dpiBA* operon induction (Miller et al., 2004)	
Cefsulodin	PBP1a and PBP1b	Rcs	Rcs regulon (microarray), P*rprA-lacZ* induction (Laubacher and Ades, 2008)	Δ*rcsF* and Δ*rcsB* more sensitive than WT (Laubacher and Ades, 2008)
Mecillinam + cefsulodin	PPB1a and PBP1b, PBP2	Rcs, Cpx, SigmaE, Bae	Rcs, Cpx, sigmaE and Bae regulons (microarray), P*rprA-lacZ* induction (Laubacher and Ades, 2008)	Δ*rcsF* and Δ*rcsB* more sensitive than WT. constitutive activation of Rcs leads to enhanced fitness (Laubacher and Ades, 2008)
Ampicillin	Multiple PBPs	Rcs, Psp, Dpi	Rcs and psp regulon induction (microarray) (Kaldalu et al., 2004), *dpiBA* operon induction (Miller et al., 2004)	Δ*dpiA* displays lower survival rates than WT (Miller et al., 2004), Δ*cpxR* is more sensitive than WT (Delhaye et al., 2016)
Penicillin G	Multiple PBPs	*Vc*Wig	Wig operon (microarray) (Dörr et al., 2016)	Δ*wigK*, Δ*wigR* lead to reduced fitness in *V. cholerae* (Dörr et al., 2016)
Multiple	Multiple	/	/	Overexpression of BaeR, RcsB, CpxR, EvgA and DcuR (and others) conferred intermediate to high level resistance to multiple beta-lactams (Hirakawa et al., 2003)
Lyzozyme	Glycan strands of PG	Rcs	P*rprA-lacZ* induction (Callewaert et al., 2009)	Δ*rcsB* and Δ*rcsF* show growth inhibition (Callewaert et al., 2009)
**MUTANTS**				
Δ*tatC*	Protein secretion and indirectly cell division	Rcs, Psp	Rcs and psp regulon induction (microarray) (Ize et al., 2004)	/
*ftsI^*ts*^*	PBP3	Dpi	*dpiBA* operon induction (Miller et al., 2004)	/
Δ*PBP4*, Δ*PBP5*, Δ*PBP7*, Δ*ampH*	Carboxypeptidase and endopeptidases	Rcs, Cpx	P*rprA-sfgfp* induction, P*cpxP-luxCDABE* induction (Evans et al., 2013)	/

*In blue, stresses that mostly target elongation processes, in green, stresses that mostly target division processes. /, no data available*.

## Transcriptomic Studies Showed That the Main ESRS of *E. coli* Can be Induced by PG-Targeting Antibiotics

Transcriptional and transcriptomic studies investigate the effect of a specific stimulus on gene expression. These types of studies were the first to demonstrate that PG-related stress could set off the main ESRS of *E. coli*. An early transcriptional study found a link between the Rcs phosphorelay and inhibition of cell wall synthesis (Sailer et al., 2003). Indeed, treatment with β-lactams induced the expression of the genes involved in the synthesis of colanic acid, a polysaccharide found in the capsule of *E. coli* (the *cps* genes), known to be regulated by the Rcs phosphorelay (Bury-Moné et al., 2009; Clarke, 2010). In contrast, antibiotics that targeted DNA replication or protein synthesis had no such effect. Surprisingly, some β-lactams were effective in triggering capsule synthesis (such as cephaloridine), while others were not (such as penicillin G), indicating that the Rcs phosphorelay could potentially sense the inhibition of a specific step in PG synthesis, and not a general inhibition of all growth-related processes (Sailer et al., 2003). In a subsequent study using transcriptomics, ampicillin, a non-specific β-lactam antibiotic that targets several PBPs, was shown to upregulate not only the colanic acid synthesis genes but also members of the Psp regulon, hinting that cell wall damage could potentially elicit multiple responses (Kaldalu et al., 2004). In a third study, the authors disrupted the twin-arginine transport (Tat) pathway, a secretion system that transports folded proteins across the IM and showed that this led to the upregulation of genes of the Rcs and Psp regulons. Although the mechanism remains unknown, it is possible that Rcs and Psp induction was triggered by the inhibition of cell division and PG hydrolysis that occurs when Tat is perturbed. Tat is indeed required for the export of the cell wall amidases AmiA and AmiC involved in division (Bernhardt and De Boer, 2003; Ize et al., 2004; Uehara et al., 2010). In a different study though, inhibiting cell division with aztreonam, a drug that specifically inhibits the septal PG synthase PBP3, resulted in very little changes in gene expression, apart from one upregulated gene involved in colanic acid production (*wcaE*) and known to be under the control of Rcs (Arends and Weiss, 2004). The last transcriptomic study reviewed here found that treatment with different combinations of β-lactams elicited as many as 4 of the 5 main ESRS of *E. coli*. Indeed, β-lactams specifically targeting the bifunctional PBPs (PBP1a and PBP1b, cefsulodin) or the monofunctional PBP2 (mecillinam), used in combination, increased the expression of genes regulated by the Rcs, Cpx, σ^E^, and Bae systems. Interestingly, Rcs was the only response that was activated in all conditions tested (multiple combinations of the drugs), suggesting that it may have an especially important role to play during PG stress (Laubacher and Ades, 2008). This is a striking example that the main ESRS of *E. coli* are turned on when PG synthesis is perturbed.

Transcriptomic studies are very informative, as they reveal a broad scope of the bacterial response to a specific stimulus. However, when it comes to the main question of this review, i.e., how do Gram-negative bacteria sense and respond to PG-related stress, they have a few shortcomings. First, these studies typically generate large amounts of data that often need to be confirmed individually (Rockett and Hellmann, 2004; Dallas et al., 2005). Second, experimental conditions such as the type and concentration of drug used, time of treatment and type of growth medium tend to vary between studies, which sometimes leads to divergent conclusions. For example, one study concluded that ampicillin did not affect capsular synthesis (Sailer et al., 2003), while another showed that ampicillin was effective in triggering the expression of the *cps* genes (Kaldalu et al., 2004). This discrepancy can easily be explained by the fact that these studies used different concentrations of ampicillin (3.75 μg mL^−1^ vs. 100 μg mL^−1^). Third, the expression of many genes is controlled by more than one stress response. For instance, *degP*, encoding the primary periplasmic protease, is induced both upon triggering of either Cpx or σ^E^ (Bury-Moné et al., 2009; Grabowicz and Silhavy, 2016) and therefore increased expression of that gene could ambiguously reflect the activation of either or both ESRS. To address this issue, it is possible to use specific reporters for different ESRS. Here a reporter protein such as β-galactosidase, luciferase or a fluorescent protein is fused to the promoter of a gene whose expression is strictly controlled by a single regulator. For example, a P*cpxP-lacZ* fusion is a specific reporter of Cpx activation (DiGiuseppe and Silhavy, 2003; Hunke et al., 2011), while a P*rprA-lacZ* fusion is a specific reporter of Rcs activation (Majdalani et al., 2002). In the next section, we cover data that result from the use of more targeted approaches to dissect the sensing of PG-related stress.

## ESRS Can be Induced Specifically by Different Types of Cell Wall Attacks

Using a specific reporter, some of the large-scale transcriptomic studies could be validated (Laubacher and Ades, 2008). The activation of the Rcs phosphorelay was verified using a P*rprA-lacZ* fusion after treatment with cefsulodin, mecillinam or both. The Rcs system was shown to be active in all 3 conditions, and this activation was dependent on the presence of the accessory lipoprotein RcsF (Laubacher and Ades, 2008). Another work with the same reporter additionally found that A22, a drug that targets MreB, an essential component of the elongation process, could also specifically elicit the Rcs response in an RcsF-dependent manner (Cho S.H. et al., 2014). Similar results were also obtained for the Cpx system using a P*cpxP-lacZ* reporter. Here, mecillinam, A22, and cephalexin (a drug targeting PBP3, essential for division) led to a 2-fold increase in Cpx activation (Delhaye et al., 2016). In addition to external stimuli, endogenous signals, such as genetically blocking a step in PG synthesis, can also set off ESRS. In *E. coli*, the deletion of a precise combination of PBPs, one carboxypeptidase (PBP5) and 3 endopeptidases (PBP4, PBP7, and AmpH) led to a reduction in motility that was dependent on the activation of both the Cpx and Rcs systems (measured with specific reporters) (Evans et al., 2013). Surprisingly the activation of the Rcs system was dependent on the activation of the Cpx system, but not *vice versa*, highlighting the existence of complex interconnections between stress responses that remain to be investigated. The reduction in motility was only observed when this specific set of genes was missing out of the 60 mutants (lacking between 1 and 7 PBPs) that were tested. This high specificity of sensing suggests that these ESRS might respond to subtle changes in PG structure and/or composition (Evans et al., 2013). Interestingly, endogenous signals do not always corroborate results obtained with the use of antibiotics. For example, whereas antibiotics with a high affinity for PBP1a led to a high induction of the *cps* genes, this was not observed in a strain in which the gene encoding PBP1a was deleted (Sailer et al., 2003). Antibiotics may thus have other effects beyond the simple inhibition of a specific enzymatic activity, such as causing a futile cycle of PG synthesis and degradation, as suggested previously (Cho H. et al., 2014). Conversely, full gene deletions may lead to polar effects or phenotypes due to the absence of the protein itself, beyond the loss of its activity. Thus, it is important to combine experiments using antibiotic treatment and genetics to firmly conclude that problems in PG synthesis are sensed by signal transduction systems. Another interesting feature is that the sensing of PG stress by ESRS is not limited to the inhibition of PBPs, as it was found that lysozyme-treated cells specifically induced the Rcs reporter (Callewaert et al., 2009). The fact that the Rcs phosphorelay is activated by antibiotics targeting PBPs, which synthesizes the PG, and lysozyme, which degrades the disaccharide backbone of the PG, is an intriguing example that this system can respond to different types of PG stress. Of note, copper ions have recently been shown to specifically inhibit L,D-transpeptidases, leading to increased sensitivity to β-lactams (Peters et al., 2018). It is well-known that treatment with high concentration of metal ions such as copper, zinc or tungstate can induce the Cpx and/or Bae response (Guest and Raivio, 2016), but a functional link between metal ions, PG damage, and activation of ESRS is still missing and could be the focus of future research.

In summary, transcriptomic studies have revealed that antibiotics targeting PBPs lead to the expression of genes controlled by the main ESRS of *E. coli*. Some of these results have since then been confirmed by more targeted approaches using specific reporters of stress responses. Although the Rcs and the Cpx systems appear as the most responsive ESRS, PG stress seems to elicit a global response through multiple regulators. It is still unclear if specific steps of PG synthesis are sensed by specific ESRS, or if all ESRS can sense a global inhibition of cell wall synthesis. Some results suggest that the former hypothesis is more likely since antibiotics that target different PBPs have a different effect on gene expression. Systematic studies of the effect of disrupting each step of cell wall synthesis and remodeling on specific reporters of the main ESRS are necessary to dissect the complicated issue of cell wall defects sensing by ESRS.

## ESRS Contribute to Fitness in PG Damaging Conditions

If the main ESRS of *E. coli* can sense damages to the cell wall, it seems reasonable to assume that these ESRS can provide a beneficial response and ensure cellular fitness when damage occurs. There are indeed a few occurrences in the literature that can clearly link the activation of a stress response to improved fitness during PG-related damage. For instance, genetically blocking Rcs induction led to increased sensitivity to lysozyme (Callewaert et al., 2009). Likewise, strains that are unable to elicit the Rcs response (Δ*rcsB*) could not grow on medium containing sublethal concentrations of cefsulodin, mecillinam or both (Laubacher and Ades, 2008). Moreover, a strain with a constitutively active Rcs phosphorelay survived better on mecillinam, or mecillinam and cefsulodin together (but not on cefsulodin alone), than the wild-type control (Laubacher and Ades, 2008). In these conditions, the survival of the cells (along with stress-sensing by Rcs), was dependent on the presence of RcsF, like most Rcs-inducing cues, but independent of RcsA (Laubacher and Ades, 2008). Since RcsA is required to activate capsule production, the increased survival was thus not dependent on the presence of a potentially protective capsule, but on other factors controlled by the Rcs response. Analogous results were obtained for the Cpx system: the growth inhibition in presence of mecillinam (as measured by a disk diffusion assay) was stronger for a strain deficient in Cpx activation (Δ*cpxR*) and slightly lower for a strain in which Cpx was turned on at moderate level. Curiously, high Cpx activation led to increased growth inhibition, indicating that the Cpx system may regulate components that are essential for cell wall homeostasis and that the extent of the Cpx response is associated with distinct effects on the PG (Delhaye et al., 2016).

To put these results in perspective, it is interesting to mention an earlier study that reported comparable findings on a larger scale. The 32 putative response regulators of all TCS in *E. coli* were overexpressed to elicit their cognate responses, and then the susceptibility of these cells against multiple β-lactams was assayed (Hirakawa et al., 2003). While there are some caveats to this method, because overexpressing the response regulator does not always lead to full activation of the TCS (Bury-Moné et al., 2009), this work helps to understand the degree at which signal transduction systems can impact resistance to β-lactams. This study revealed that the overexpression of 13 response regulators led to increased resistance to several β-lactams, as indicated by a higher minimal inhibitory concentration (MIC). Those response regulators included BaeR and RcsB, which conferred high to intermediate resistance and CpxR, which conferred low-level resistance. The other response regulators that provided high-level resistance were EvgA and DcuR (Hirakawa et al., 2003). While the EvgAS system controls the expression of a multidrug transporter (Nishino and Yamaguchi, 2002) and can therefore logically be linked to survival in the presence of β-lactams, the relationship is more cryptic in the case of the DcuRS system, which controls the expression of genes related to the intake and metabolism of external C4-dicarboxylates (Golby et al., 1999). Clearly resistance to (and most probably sensing of) cell wall defects is also dependent on other, perhaps less studied, signal transduction systems that have previously not been linked with envelope quality control and monitoring.

## The Regulon of Select ESRS Include Genes Encoding Cell Wall Modifying Enzymes

One would expect that when the wall is attacked, the cell responds either by increasing the amount of the building machineries that are inhibited or by diverting resources to increase the expression of alternative machineries to reinforce the cell wall. Yet while the main ESRS seem to be able to sense PG stress, and at least two of them (the Rcs and the Cpx responses) have been shown to increase *E. coli* survival during cell wall targeting antibiotic treatment (as elaborated in earlier sections), their response is usually thought to deal with general quality control of the envelope and thus with effects that are not directly involved in PG synthesis, its regulation or its protection. In other words, the presence of a feedback loop that induces the production of new PBPs or other cell wall altering enzymes or protective agents in response to sensing cell-wall defects by ESRS has seldom been demonstrated. Nevertheless, other examples have been reported. First, the Rcs phosphorelay is active during treatment with lysozyme and induces the expression of two lysozyme inhibitors, *ivy* and *ydhA*, which are responsible for better survival during lysozyme treatment (Callewaert et al., 2009). A more noteworthy instance concerns the Cpx system. Relatively recent studies found that genes encoding 3 predicted cell wall modification proteins were part of the Cpx regulon: Slt, a lytic transglycosylase, LdtD, a L,D transpeptidase that catalyzes non-canonical 3–3 L,D crosslinks between glycan strands in the PG, and YgaU, a conserved protein with a LysM domain found in enzymes that interact with and degrade the cell wall (Raivio et al., 2013; Bernal-Cabas et al., 2015). The Cpx-dependent expression of *ldtD* was later shown to substantially influence PG-related processes such as elongation and division as well as sensitivity to β-lactams, as *ldtD* was largely responsible for cell wall defects observed in conditions that induce a very high level of Cpx response (Delhaye et al., 2016). In contrast, a moderate Cpx-induced expression of *ldtD* may also explain the Cpx-dependent increased survival during PG stress (Delhaye et al., 2016) (summarized in Table 1), as it has been shown recently that production of LdtD along with surprisingly few additional factors lead to a complete bypass of D,D transpeptidase activity of PBPs for cell wall synthesis, and broad-spectrum β-lactam resistance (Hugonnet et al., 2016). While it seems that depending on the condition (expression levels for exemple), LdtD may have either a beneficial or detrimental effect, it is clear that its expression impacts PG synthesis. These data showed that at least one of the main ESRS of *E. coli* can modulate the expression of cell wall acting enzymes, and the integrity of the PG itself. As the Cpx system is known to be active during late exponential and stationary phase, this has important implications for the regulation of cell wall structure in response to stress, but also for housekeeping purposes. It should be noted that Rcs has been reported to modestly increase (around 2-fold) the expression of *mrcA, mrcB* (the genes encoding PBP1a and PBP1b) and *minD* (a cell division inhibitor) in a transcriptomic study (Ferrières and Clarke, 2003). As far as we know, it is the only mention of these enzymes being in the regulon of the Rcs response. It also has not been tested whether activation of Rcs could lead to cell wall alterations *via* these enzymes, although it was shown that *E. coli* requires Rcs to regenerate its cell wall *de novo* after it was completely removed with lysozyme (Ranjit and Young, 2013). Additionally, Rcs was shown to promote the expression of both *ftsA* and *ftsZ*, which are genes that are crucial for cell division (Carballès et al., 1999). However, here again, no functional link was established between the activation of Rcs and alterations in cell division. These data suggest that, similarly to Cpx, Rcs may influence cell wall synthesis and housekeeping, but more research is necessary to understand how this function may be accomplished.

## Unexpected Signal Transduction Systems Can Monitor Growth Processes

**Figure 3:**
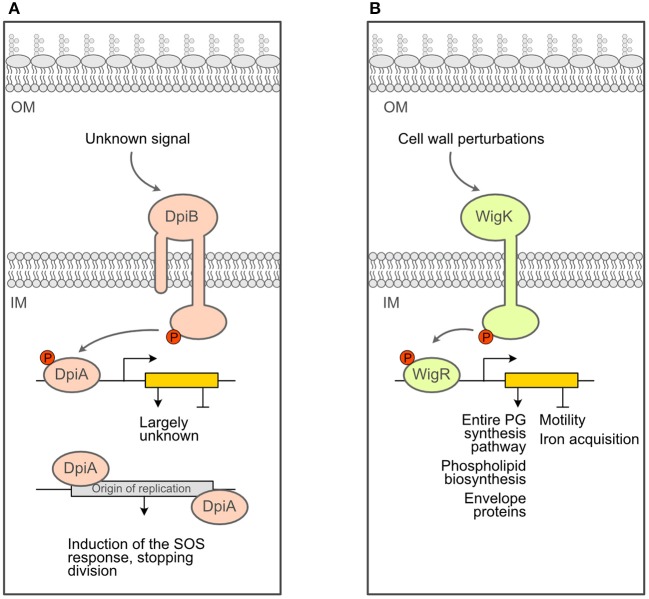
Schematics of additional response systems that deal with PG stress. **(A)**. Schematics of the DpiBA two-component system. **(B)** Schematics of the WigKR two-component system of *Vibrio cholerae*.

There are additional clues in the literature that sensing and responding to PG stress is not an activity limited to the main ESRS described above. For instance, several lines of evidence connect PG synthesis during cell division with the DpiBA two-component system (schematized in Figure 3A), although this system was primarily associated with DNA replication and induction of the SOS response: when overexpressed, DpiA, the effector protein (or response regulator) of the TCS, binds replication origin sequences on the *E. coli* chromosome and certain plasmids, which interferes with DNA replication and triggers the SOS response (Huisman et al., 1984; Ingmer et al., 1998; Miller et al., 2003). First, it was found that treating cells with ampicillin, cephalexin or pipericillin (which targets PBP3) turned on the expression of both the *dpiBA* operon and *pabA*, a gene regulated by DpiA. Second, inactivation of PBP3 by shifting an *ftsI*^*ts*^ (encoding PBP3) strain to non-permissive temperature and therefore blocking cell division also resulted in induced *dpiBA* expression. Interestingly, no effect was observed when PBP2 and FtsW were inactivated, indicating that the lack of PBP3 activity is a specific stimulus for *dpiBA* expression (Miller et al., 2004). Third, treatment with ampicillin and inactivation of PBP3 both activated the expression of *sfiA*, an SOS response-induced gene that prevents FtsZ polymerization and thus cell division in a *dpiA*-dependent manner. Taken together, these data suggest that interfering with PG assembly, in particular during cell division, triggers the DpiBA two-component system. Supporting the physiological relevance of these findings, *dpiA* null mutants display markedly reduced cell survival when exposed to ampicillin for a short time (<4 h) (Miller et al., 2004). However, it remains unclear whether DpiA acts alone as an effector protein to set off the SOS response during treatment with β-lactams, or if genes present in the DpiBA regulon are also necessary for resistance to β-lactams.

Another example of non-canonical ESRS being able to sense and respond to cell wall defects is the newly characterized TCS WigRK described in *Vibrio cholerae* (schematized in Figure 3B). It was identified in a Tn-Seq screen for *V. cholerae* factors that are required for recovery from penicillin exposure (Dörr et al., 2016). Mutants that lack *wigK, wigR* or *wigRK* exhibit lower (2–3 orders of magnitude) colony-forming units after treatment with penicillin G. Extraordinarily, the regulon of this TCS includes the full set of genes required for cell wall biosynthesis (Dörr et al., 2016). The increased expression of these genes leads to a higher cell wall content and markedly increased resistance to hypo-osmotic shock (Dörr et al., 2016). Interestingly, disrupting cell wall synthesis with penicillin induces the expression of *mraY* (involved in lipid II biosynthesis), *murJ* (lipid II flippase) and genes encoding PBP1A and PBP1B (the major *V. cholerae* cell wall synthases) in a *wigR-*dependent manner. In contrast, compounds targeting envelope processes unrelated to cell wall synthesis, such as cerulenin (inhibitor of fatty acid synthesis) and crude bile (general membrane perturbator), did not result in induction of *pbp1a*, suggesting that WigKR is turned on in response to cell wall damaging agents and not cell envelope damage in general. In addition to the important role of this system in surviving treatment with cell wall inhibitors, WigKR also affects cell wall homeostasis during normal growth. Indeed, mutants lacking *wigR* had a larger diameter and cell volume, whereas strains overexpressing WigR had a significantly reduced cell width, indicating a fundamental role of this TCS in maintaining cell wall homeostasis (Dörr et al., 2016).

## The Mechanisms of Sensing Cell Wall Damage by ESRS Are Largely Unknown

There is convincing evidence that ESRS can sense and respond to cell wall damage, yet the molecular signals that trigger these responses remain mostly unknown. One possibility is that ESRS actually sense downstream effects of cell wall impairment, such as membrane perturbations (known to trigger the Rcs response, Farris et al., 2010) due to cell shape defects (Huang et al., 2008). However, there is some evidence that suggests that the signal sensed could also be direct and specific, notably the fact that β-lactams with different targets elicit different responses (Sailer et al., 2003; Arends and Weiss, 2004; Kaldalu et al., 2004; Laubacher and Ades, 2008). Some studies suggest that the main candidates for a direct and specific sensing are the pool of PG precursors and PG fragments, destined for recycling (Sailer et al., 2003; Evans et al., 2013; Dörr et al., 2016). Such a signal has already been described for the regulation of the production of β-lactamase (Jacobs et al., 1994, 1997). In many Gram-negative bacteria, the expression of the β-lactamase AmpC is induced by the AmpR regulator after activation by β-lactams (Lindberg et al., 1985; Vadlamani et al., 2015). The activity of AmpR is modulated by PG intermediates: it is maintained in its inactive form by a UDP-MurNAc-pentapeptide, a PG precursor whose concentration decreases during treatment with β-lactams, and activated by a anhMurNAc-tripeptide, a product of PG recycling that accumulates in the cytoplasm during treatment with β-lactams (Jacobs et al., 1997). So not only does treatment with β-lactams lead to changes in the pool of different PG species and intermediates, but these changes have been demonstrated to influence the activity of a very specific response system that directly deals with the initial stress. This is an elegant mechanism, and there is supporting evidence that a similar process could be responsible for the activation of ESRS during cell wall stress. For instance, an *E. coli* mutant strain that lacks 4 specific PBPs has constitutively active Rcs and Cpx systems (as discussed previously), and the amount of pentapeptides and different species of cross-linked muropeptide was shown to rise and fall along with the activity of the Cpx and Rcs systems (Evans et al., 2013). Still, a detailed mechanistic understanding of how the main ESRS, as well as other signal transduction systems, can sense cell wall damage, remains elusive.

## Conclusion

*E. coli* and other Gram-negative bacteria are equipped with sophisticated systems (including ESRS) to monitor and convert a stress stimulus into remodeling their gene expression pattern, thereby rewiring the cell physiology to match the new environmental state. While important envelope biogenesis processes have been shown to be monitored by precise signal transduction systems, the question of how cell wall related processes, such as elongation and division, are tracked to avoid lethal malfunctioning, remains unresolved. Extensive research efforts were focused on identifying the players required for PG synthesis and its control in *E. coli* and other species. For example, post-translational regulators were discovered, such as the lipoproteins LpoA and LpoB, which modulate the activity of the PG synthases PBP1a and PBP1b, respectively (Paradis-Bleau et al., 2010; Typas et al., 2010). However, few transcriptional regulators of PG synthesis are actually described in *E. coli*. This review attempts to shed light on these two issues: how can stress responses sense the correct or incorrect synthesis of the cell wall (sensing) and how do they modulate gene expression to respond to any defects detected (response)?

Concerning the sensing, the body of work presented here clearly outlines the fact that the main ESRS of *E. coli* can sense a compromised cell wall. Both exogenous factors (such as treatment with β-lactams or lysozyme) and endogenous factors (such as the deletion of a specific set of PBPs) can act as a trigger to set off the Rcs, Cpx, Bae, σ^E^, or Psp response. Depending on the stimulus, one or multiple responses can be fired off simultaneously. Likewise, a specific stress response can be triggered by one or multiple stimuli. This highlights a major lack of knowledge: whereas the sensing of PG stress by major ESRS has been documented numerous times in the literature, the mechanistic details of such sensing by the different stress responses are often missing. Moreover, as this has not been the focus of intense research, there are probably many occurrences of sensing of cell wall defects by signal transduction systems that remain to be revealed, both by well-known ESRS and by other, less-studied systems.

Regarding the response, a few signal transduction systems have been shown to increase survival when the integrity of the PG is challenged. These include some of the main ESRS, Rcs, Cpx, and Bae, as well as other TCS, such as EvgA and DcuR. In most cases, it is still unclear how activation of these responses helps cells with cell wall defects. Do they deal with side-effects of PG synthesis inhibition by stabilizing other components of the envelope? Do they directly regulate growth to adapt to certain PG stress? Or is it a combination of both? So far, only two of the main ESRS, Rcs and Cpx, have been shown to not only detect cell wall perturbations but also to control the expression of genes involved in PG remodeling in *E. coli*. While a functional link between the activation of Cpx and growth-related processes could be described, this is not the case for Rcs.

Future research should focus on these shortcomings (concerning the mechanisms of sensing and response), to elucidate how cells react to harsh stresses such as cell wall-targeting antibiotic treatment, but also how they adjust their cell wall to different growth conditions, for example when switching to stationary phase or during infection. To this end, complementary approaches could be envisioned. First, a global, high throughput approach may help to thoroughly define the stimuli triggering each ESRS and other signal transduction systems. For instance, a library of strains could be engineered to carry specific reporters of these systems; the activity of these reporters could be quantified when cells are grown in a vast array of conditions known to perturb PG integrity, hence providing a systematic overview of which PG stress induces which pathway. Besides, a more directed genetic approach could help identifying novel factors involved in sensing PG stress, for example by screening a mutagenized library to identify genes that are required for the activation of a given ESRS by a specific PG-damaging condition. A follow-up biochemical characterization of the newly identified factors would be needed to uncover the molecular mechanism(s) of PG stress sensing by ESRS. Moreover, these strategies can be combined with the analysis of PG species released under different stress conditions to potentially identify PG fragments that could act as inducers or repressors of stress responses.

An as yet largely untapped resource for insights might be found in the many stress responses that were not previously thought to deal with envelope perturbation, including those that are not as extensively investigated as the main ESRS of *E. coli*, as it is likely that those systems still conceal interesting secrets. A better knowledge of how stress responses can sense and mitigate PG stress can lead to a better understanding of both the functioning of stress responses and the regulation of cell wall synthesis.

## Author Contributions

AD, GL, and J-FC wrote the manuscript.

### Conflict of Interest

The authors declare that the research was conducted in the absence of any commercial or financial relationships that could be construed as a potential conflict of interest.
